# Peritonitis and Its Treatment

**Published:** 1908-01

**Authors:** 


### PERITONITIS AND ITS TREATMENT.

---

Berry in the Lancet, September 7th, says that underlying the treatment of all forms of peritonitis there are certain broad principles that should guide us to the best possible treatment in the individual cases. The reason that inflammation of the peritoneum is so much more dangerous than inflammation of other organs or tissues is that the peritoneal cavity is a vast lymphatic space, from which absorption of toxins readily takes place. By peritonitis is meant the inflammation of the peritoneum produced by the presence of bacteria, either from the blood stream, as in the course of an acute septic lesion of some other part of the body, or by direct introduction from without as from a wound. In the majority of cases of acute appendicitis, the amount of poison that escapes is small and can be dealt with by the peritoneum itself, which is the reason that most cases of acute or subacute appendicitis recover spontaneously. In the case of a perforation of a large hollow viscus like the stomach or cecum, however, preforation is rarely preceded by adhesive inflammation, so that there is frequently poured out a vast quantity of septic material, much greater when the perforation occurs during digestion. A sudden invasion of the peritoneal cavity by bacteria results in a rush of blood to the affected area. The abdominal viscera and the great omentum are engorged with blood, so that other parts of the body are greatly deprived of it. Berry lays special stress on this as an important factor in the cause of the shock and feeble rapid pulse often met with in peritonitis and states that the proper mode of treatment is to replenish the empty vessels by giving the patient plenty of fluid. In regard to the effusion of serous fluid into the peritoneal cavity, occurring in so many cases of acute peritonitis, Wright has shown that the bactericidal power of serous fluid diminishes in the immediate neighborhood of an affected area, and becomes exhausted. The letting out this fluid by operation leads to effusion of further amount of fresh serum, and destruction of more bacteria; hence the beneficial effect of drainage of inflamed areas. The beneficial effects of draining away the fluids in chronic tuberculous peritonitis are thus to be explained. He further points out that the effect of a dose of toxin on the opsonic power of the blood plasma is a lowering of the opsonic index—the induction of the negative phase. The addition of a traumatism at this stage means a further dose of toxin, so that the negative phase may be increased even to death. By waiting a few days, however, the opsonic index is greatly raised above normal, and operations can be performed with greater safety. As regards the question of the poison, Berry thinks that of late undue stress has been laid on the quality of the bacteria and too little on the quantity of poison in the case of a perforative appendicitis, and that it is on the quantity rather than on the quality, that the severity of the attack depends, especially in peritonitis associated with appendicitis. In connection with acute appendicitis there is actual or visible perforation only in a minority of cases. In support of this he quotes Ochsner's statistics. Berry is opposed to the view of operating always. as soon as the patient is seen, for in this case we should very often operate on patients at an unfavorable stage when mortality is high, and it is usually best to defer operation. A little later, intraperitoneal inflammation may have aided by shutting off and localizing the poison.

It is absurd to pretend that operations, especially major operations, on patients suffering from acute peritonitis do not carry with them much risk. The question is whether the risk of the operation, with its necessary attendant evils, is or is not more than counterbalanced by the good it may do. Shock from manipulation and further absorption of poisons are the evils that may ensue from a large operation. The good thereof consists in closing the perforation or removing a septic focus to prevent further entrance of poison, and removing, to only a limited extent, however, poison from the peritoneal cavity. Poison can not be removed from the blood, but by encouraging excretion mainly by the administration of fluid the blood can be helped to get rid of it. Berry divides all cases of peritonitis into four classes: 1. Those in which there is no perforation of any viscus and no collection of fluid in the peritoneal cavity. The amount of poison is necessarily small in the peritoneal cavity, and eliminant treatment by free purgation with salines, and administration of large quantities of fluid by the mouth, rectum, or intravenously is the chief indication. Opium should be avoided. 2. Cases in which we know or suspect that there is a recent perforation of a hollow viscus, such as the stomach or intestine. Here immediate operation should be performed with the object of closing the perforation and preventing further extravasation into the peritoneal cavity. The prevention of peritonitis, rather than its cure, is aimed at. 3. This important and numerous class includes the majority of cases of appendicitis. There is considerable peritonitis, involving much of the peritoneal cavity, with more or less tendency to the concentration of the inflammation in one lesion. Septic fluid is more or less localized. The patient's condition will depend on the extent to which the septic focus is shut off by omentum and adhesions, and to the absence of great tension. In such a case the operation for its removal may be far more dangerous than leaving a septic focus where it is; at any rate, for a short time, until a more favorable period for operation. Ochsner's opinions and statistics support this conclusion. In the presence of such cases we should ask ourselves, (a), What is likely to be the course of events if we do not operate at once? (b), What good and what harm may we do by operating at once? (c), If we operate, what operation should be adopted? Berry discusses these three questions fully. He opens localized collections of pus, when sufficiently large, through the posterior fornix in women, and through the rectum in men. Great care must be taken to protect non-adherent peritoneal cavities. 4. In the fourth class there is a general diffuse peritonitis, with general distension of the whole abdomen, and purulent fluid diffused over a large portion or the whole of the abdomen. There is much difference of opinion regarding the appropriate treatment of this condition. The first plan is to keep the patient quiet and the stomach empty until the inflammatory processes and exudates have become localized, and if the patient lives until this has taken place, then to operate. The next method consists in freely opening the peritoneal cavity, breaking up adhesions, opening pockets, and irrigating the entire peritoneum. The third method includes opening the abdomen, removing the cause of inflammation, and thorough drainage with the least possible disturbance of viscera.

Berry, having tried all three methods, believes that the Ochsner method is the most suitable for most cases of diffuse peritonitis. For the very worst class, Murphy's method of small abdominal opening, the closing of the perforation and the removal of the appendix, the introduction of a large drainage tube, with the patient in a sitting posture, and the administration of salt solution by rectum every two hours is good. He agrees with those who condemn the very large operations, the mortality of which has been terrific. He notices a tendency among surgeons of large experience to abstain more and more from such operations. He is greatly impressed with the advantages of a sitting posture, advocated by Murphy, and tries to persuade all who send cases of peritonitis to the Royal Free Hospital to adopt that precaution. Finally, he says: "It may seem to you an extraordinary thing for me to say, but I say it with deliberation and with a profound conviction of its truth, that the very high gross mortality in acute appendicitis, which undoubtedly exists in this country (and in other countries as well) is largely due to the fashion of indiscriminate, excessive and injudicious operating, which not long ago was at its height, but which I venture to think and hope is now showing signs of subsiding." Again: "It is not unusual to hear it stated that modern operations for appendicitis have resulted in a great saving of life. I believe that on the whole the exact converse is the case." He believes that a return to the old treatment of appendicitis in vogue twenty-five years ago would result in a lesser mortality. He does not refer to operations done by surgeons of large experience, but rather to the far larger number of operations performed by those who do not publish their results, and who do not discriminate between cases in which operation is indicated and those in which it is not.—*Jour. A. M. A.* Oct. 5th. '07.

